# (Pro)renin Receptor and Blood Pressure Regulation: A Focus on the Central Nervous System

**DOI:** 10.2174/1570162X20666220127105655

**Published:** 2022-11-23

**Authors:** Lucas A.C. Souza, Yumei Feng Earley

**Affiliations:** 1 Departments of Pharmacology and Physiology & Cell Biology, University of Nevada, Reno, School of Medicine, Reno, NV, USA;; 2 Center for Molecular and Cellular Signaling in the Cardiovascular System, University of Nevada, Reno, Reno, NV, USA

**Keywords:** (Pro)renin receptor, brain, central nervous system, blood pressure, hypertension, RAS

## Abstract

The renin-angiotensin system (RAS) is classically described as a hormonal system in which angiotensin II (Ang II) is one of the main active peptides. The action of circulating Ang II on its cognate Ang II type-1 receptor (AT_1_R) in circumventricular organs has important roles in regulating the autonomic nervous system, blood pressure (BP) and body fluid homeostasis, and has more recently been implicated in cardiovascular metabolism. The presence of a local or tissue RAS in various tissues, including the central nervous system (CNS), is well established. However, because the level of renin, the rate-limiting enzyme in the systemic RAS, is very low in the brain, how endogenous angiotensin peptides are generated in the CNS—the focus of this review—has been the subject of considerable debate. Notable in this context is the identification of the (pro)renin receptor (PRR) as a key component of the brain RAS in the production of Ang II in the CNS. In this review, we highlight cellular and anatomical locations of the PRR in the CNS. We also summarize studies using gain- and loss-of function approaches to elucidate the functional importance of brain PRR-mediated Ang II formation and brain RAS activation, as well as PRR-mediated Ang II-independent signaling pathways, in regulating BP. We further discuss recent developments in PRR involvement in cardiovascular and metabolic diseases and present perspectives for future directions.

## 1 INTRODUCTION

The renin-angiotensin system (RAS) is a much more complex system than originally envisioned, comprising different peptides, enzymes, and receptors with endocrine, paracrine, and autocrine characteristics [1]. The importance of this system in the pathogenesis of hypertension and other cardiovascular diseases is exemplified by the therapeutic success of angiotensin converting enzyme (ACE) inhibitors and angiotensin II type I receptor (AT_1_R) antagonists in treating these diseases [2, 3]. As classically described, the RAS includes angiotensinogen, an α-globulin protein produced mainly by the liver that is cleaved in the circulation by the enzyme renin (produced by juxtaglomerular cells of the kidney) to form the decapeptide angiotensin I (Ang I). Ang I is then cleaved by ACE, an enzyme mainly produced by endothelial cells in the pulmonary circulation and the kidneys, forming the octapeptide angiotensin II (Ang II). Ang II is considered the main biological peptide of the RAS, and its actions, which can lead to the pathogenesis of hypertension through sodium retention [4, 5], vasoconstriction [6], aldosterone synthesis and secretion from the adrenal cortex [7-9], increased sympathetic activity [10, 11] and thirst [12, 13], are attributable to binding of this peptide to angiotensin receptors [14-16].

In addition to the classical systemic RAS, the local or tissue RAS in various organ systems, including the brain, is now well established. However, because the level of renin, the rate-limiting enzyme in the systemic RAS, is very low in the brain, how endogenous angiotensin peptides are generated in the central nervous system (CNS) has been the subject of considerable debate and is the focus of this review. Here, we discuss the brain prorenin-angiotensin system, with an emphasis on the identification of the (pro)renin receptor (PRR) as a key component of the brain RAS in the production of Ang II in the CNS. We highlight the cellular and anatomical locations of the PRR in the CNS and summarize studies using gain- and loss-of function approaches to elucidate the functional importance of the brain PRR in blood pressure (BP) regulation through Ang II formation and brain RAS activation as well as through Ang II-independent signaling pathways. We further discuss recent developments in elucidating the role of the PRR in cardiovascular and metabolic diseases and present perspectives for future directions.

## 2 RAS IN THE CNS

Blood-borne substances, including Ang II, can act on highly vascularized structures of the CNS located around the ventricles, which are characterized by the lack of a blood-brain barrier (BBB). These circulation-accessible structures, termed circumventricular organs [17, 18], include the subfornical organ (SFO), organum vasculosum of the lamina terminalis (OVLT), and area postrema (AP) [19], as illustrated in Fig. (**1**). Because many studies have demonstrated expression of RAS components in areas of the CNS that are protected by the BBB, and neither renin [20] nor Ang II [21] can penetrate the BBB under physiological conditions, it was reasonable to hypothesize that the CNS is capable of producing Ang II locally. Several pathways for Ang II generation in the CNS have been proposed. For example, Ang II can be produced by the conversion of angiotensinogen to Ang I by cathepsins D and E, and elastase and proteinase 3, or directly by the conversion of angiotensinogen to Ang II by cathepsin G, tonin, elastase, and proteinase 3 [22, 23]. More recently, the PRR, possibly by activating prorenin to cleave angiotensinogen to Ang I, has been shown to mediate Ang II formation in the CNS [24, 25]. Under hypertensive conditions, the BBB is disrupted in association with inflammation, oxidative stress, and the release of vasoactive molecules in the brain microvasculature [26]. In hypertension, Ang II can also access areas of the CNS that are protected by a BBB, like the paraventricular nucleus of hypothalamus (PVN), nucleus tractus solitarius (NTS), and rostral ventrolateral medulla (RVLM) [19, 27], as illustrated in Fig. (**1**). This BBB breakdown observed in hypertensive animals seems to be dependent, at least in part, on Ang II, as evidenced by the fact that chronic oral administration of an AT_1_R antagonist, but not a vasodilator drug, attenuates the BBB breakdown [19, 27]. However, the mechanisms responsible for BBB breakdown in hypertension remain to be elucidated.

It is now well established that most RAS components are expressed in the CNS. The first RAS component to be identified in the CNS was renin. In 1971, Ganten *et al.* [20] showed that renin enzymatic activity is approximately 10-times higher in the caudate nucleus than in mesenteric artery branches. Moreover, a number of studies have demonstrated both mRNA and protein expression of angiotensinogen [28-32], renin [28, 29, 33], ACE [34-36], AT_1_R [37-43], and AT_2_R [40, 41, 44, 45] in the CNS. AT_1_Rs are particularly highly expressed in areas of the CNS related to BP regulation and fluid homeostasis [37, 38, 40-43, 46, 47], including the OVLT, SFO, median preoptic nucleus (MnPO), PVN, supraoptic nucleus of the hypothalamus (SON), dorsomedial hypothalamic nucleus (DMH), RVLM, NTS, arcuate nucleus (Arc), and AP. AT_2_Rs are expressed in areas not traditionally related to BP regulation, such as the lateral septum, several thalamic nuclei, the subthalamic nucleus, locus coeruleus and inferior olive nucleus [37, 38, 40, 41, 44, 45], as well as in areas related to BP regulation, such as the SFO, OVLT, PVN, Arc, MnPO, DMH, NTS, AP, medial prefrontal cortex (mPFC), and amygdala [44, 47]. In general, AT_1_Rs and AT_2_Rs are expressed in separate populations of neurons within each brain region. Some regions, such as the OVLT, SFO, PVN and Arc, contain more AT_1_R-positive cells than AT_2_R-positive cells, whereas other regions, including the NTS and AP, contain more AT_2_R-positive cells than AT_1_R-positive cells [47]. In addition to components of the classical vasoconstrictor axis of the RAS, many research groups have also demonstrated that angiotensin converting enzyme 2 (ACE2) [48-51], which converts the octapeptide Ang II into the heptapeptide angiotensin-(1-7) (Ang-(1-7)), a Mas receptor agonist, is expressed and plays an important role in the CNS [52, 53]. Ang-(1-7) has effects that oppose those of Ang II, promoting vasodilation, decreasing BP, and improving baroreflex sensitivity [5, 54, 55].

## 3 RENIN AND PRORENIN IN THE CNS

Renin expression and enzymatic activity in the brain have been the subject of debate for decades. Most studies showed low level renin mRNA expression in the brain using techniques such as Northern blot hybridization [28], ribonuclease-protection [56], and competitive polymerase chain reaction (PCR) [57]. To our knowledge, two reporter mouse strains have been used to investigate renin expression and renin promoter activity in the brain [29, 58, 59]. In the first case, Lavoie *et al.* [29, 58] used a mouse strain that expresses enhanced green fluorescent protein (eGFP) driven by the renin promoter (REN-1) that allows identification of the cellular and spatial distribution of brain renin expression [60], demonstrating renin expression in different brain regions, including the SFO and RVLM. Using NeuN immunolabeling, they also found that renin was co-localized mainly with neurons and demonstrated adjacent expression of renin and angiotensinogen in the RVLM [29]. In the other case, Allen *et al.* bred a mouse strain expressing Cre recombinase under the control of the human renin promoter with Cre recombination-dependent lacZ reporter mice. They showed that human renin promoter activity is detectable in neurons in all areas investigated and found that its expression did not appear to correlate with the expression of other RAS components, such as angiotensinogen and Ang II receptors [59]. Interestingly, unlike Lavoie *et al.* [29, 58], they did not detect human renin promoter activity in the SFO or RVLM [59]. The use of different genetic strategies and renin promoters from different species in the development of these mouse strains may explain the divergent results found by the two groups. In the mouse strain developed by Lavoie and colleagues, GFP is expressed under the control of the endogenous mouse renin promoter together with intact endogenous mouse renin expression and identifies cells that currently express renin. On the other hand, the mouse strain used by Allen and coworkers expresses Cre recombinase under the control of the human renin promoter, and thus illuminates cells that have ever expressed renin. Notwithstanding these differences, the results of these studies provide evidence of endogenous renin promoter activity and expression in the brain.

In juxtaglomerular cells of the kidney, renin mRNA is translated into preprorenin starting at exon 1a (renin-a). After removal of the signal peptide, prorenin can be either directly secreted or further processed to its active form, renin, and then secreted into the circulation [61]. In the CNS, there are two alternative transcripts for the renin gene [62, 63]. Renin produced by transcription starting at exon 1a (renin-a) is the same as the key prorenin with an intact signal peptide and is predicted to be secretable into the extracellular space as prorenin. This brain isoform, also named secreted renin (sREN or more accurately sProrenin), is distinct from the intracellular form of renin [64]. Prorenin has only 3% of the intrinsic enzymatic activity of renin in plasma [65], and it is still not clear whether sProrenin can be enzymatically processed to renin in the brain [22]. However, exogenous prorenin infused into the mouse brain can mediate Ang II formation and elevate BP, effects that are strictly dependent on its receptor, the PRR, suggesting possible non-proteolytical activation of prorenin in the CNS through binding to the PRR [24].

Renin-b, a novel transcript starting at exon 1b of the renin gene that is expressed in the brain, has also been identified in rats, mice, and humans. This transcript is responsible for the translation and synthesis of a specific brain renin isoform that lacks the signal peptide and part of the prosegment, also named intracellular renin (icREN) by virtue of its intracellular localization [61-63]. To initially dissect the role of icREN in BP regulation, Lavoie *et al*. (2006) [66] generated transgenic mice in which human icREN expression is driven by the GFAP promoter and bred them with mice expressing human angiotensinogen driven by the same promoter. They found an increase in fluid intake, mean arterial pressure, and BP response to intracerebroventricular (ICV) infusion of losartan, suggesting increased brain RAS activity in these mice. Later, the same group selectively deleted icREN and surprisingly also observed a significant increase in systolic BP [67]. This effect was associated with increases in sympathetic activity to the heart and kidneys, AT_1_R expression in the PVN, brain RAS activity, and expression of total renin (icREN+sProrenin) in the RVLM [67]. They concluded that, under baseline conditions, icREN suppresses sProrenin expression in the brain, an action that is impaired by hypertension-induced stimuli. Taken together, these data indicate that, contrary to expectations, endogenous icREN might modulate the expression of sProrenin to exert a protective role. On the other hand, the direct role of endogenous sREN or sProrenin in the CNS has been explored in very few studies. In one study, Xu *et al.* investigated the role of classical sREN or sProrenin, encoded by renin-a, in cardiovascular and metabolic regulation in a mouse model in which renin-a was specifically deleted in neurons or astrocytes [68]. They found that deletion of renin-a in these cells caused no changes in BP, heart rate (HR), food or fluid intake, urinary volume, sodium intake, or parameters related to energy output under baseline conditions. The authors concluded that CNS sProrenin may not play a major role in cardiovascular or metabolic regulation. However, this conclusion was later refuted by the same group using the icREN-deleted mice described above, pointing to the potential importance of sProrenin in the neural regulation of BP and metabolic rate [67, 69]. Notwithstanding these observations, the role of CNS sProrenin in the context of cardiovascular or metabolic diseases is not yet fully understood. Considering the key role played by the brain RAS in the pathogenesis of these diseases [6, 25, 51, 70-73], future studies are warranted to fill this important knowledge gap.

Although the role of endogenous CNS sProrenin in BP regulation is not yet defined, the functional importance of exogenous prorenin in the CNS has been revealed by several studies [24, 25, 74-76]. For example, direct ICV infusion of prorenin induces an increase in BP in C57Bl/6J mice that is attenuated by PRR deletion in neurons [24] or co-administration of a PRR antagonist (PRO20) [25]. On the other hand, the pressor response to central infusion of prorenin is exacerbated by PRR overexpression in neurons [75]. Interestingly, ICV infusion of renin also elevates BP, but this effect is not attenuated by deletion of the neuronal PRR [24], suggesting that renin is capable of exerting its enzymatic activity independent of the PRR. This finding also indicates that this enzymatic activity probably does not require conversion of sProrenin to renin in the CNS, but may involve nonproteolytic activation through binding to the PRR. In a later study, Huber *et al.* [74] showed that bilateral microinjection of human prorenin into the PVN increases splanchnic sympathetic nerve activity (SSNA) in anesthetized Sprague-Dawley (SD) rats. Interestingly, co-administration of the handle region peptide (HRP), another PRR antagonist, or Tiron, a scavenger of reactive oxygen species (ROS), prevents this increase in SSNA. These observations show that exogenous prorenin acts through the PRR in the CNS to play important roles in BP and autonomic regulation.

Despite extensive literature describing renin/prorenin expression and enzymatic activity as well as increases in sProrenin levels/expression in the CNS [24, 28, 29, 56-59, 62, 63, 66-68, 71, 77, 78], the precise cellular and regional localizations as well as the physiological significance of sProrenin remains incompletely understood, inspiring a continuing debate [73, 79-84]. Ganten *et al.* (1971) [20] demonstrated renin enzymatic activity in the caudate nucleus, frontal cortex, thalamus, hypothalamus and brainstem of dogs, whereas Hirose *et al.* (1978) [85] showed renin enzymatic activity in whole-brain extracts of rats. Other studies have also demonstrated renin protein expression using immunoassays. Hermann *et al.* [86], in 1987, reported that renin is expressed in neuronal and glial cell cultures from the hypothalamus and brainstem of Wistar Kyoto (WKY) rats and that it is expressed at higher levels in cell cultures from spontaneously hypertensive rats (SHRs) than WKY rats. Moreover, renin and/or sProrenin protein levels and enzyme activity, measured as total renin (renin + prorenin) by enzyme-linked immunosorbent assay (ELISA) and enzyme kinetics assays, respectively, have been previously reported in the hypothalamus and brainstem of hypertensive DOCA-salt mice [24]. On the other hand, a recent study by van Thiel *et al*. (2017) provides an example of the opposing point of view, arguing that, because of the nanomolar-range affinity of the PRR for prorenin, higher than physiological/pathophysiological prorenin levels may be required for this binding. In addition, this study further suggests that there is no brain RAS at all [80]. They reported that 1) angiotensin-generating ability in the brain is renin-dependent, 2) DOCA-salt and Ang II hypertension suppress both plasma and brain renin, 3) angiotensinogen protein is undetectable in the brain, despite expression of angiotensinogen mRNA, and 4) the levels of renin/sProrenin are profoundly decreased (>60%) in all brain regions analyzed after washing away blood from the brain vasculature. Citing these lines of evidence, the authors raised questions about the local production of Ang II in the brain, despite the numerous studies supporting it [24, 25, 70, 71, 87-91]. It is worth noting that, although there was a significant decrease in renin/sProrenin levels after blood was removed from the brain vasculature in this latter study, renin/sProrenin was still detectable in most brain regions, showing that renin and sProrenin are indeed expressed in the brain. In support of this conclusion, a very early study by Genain *et al*. (1985) [78] examined renin enzymatic activity and angiotensinogen concentration in different brain regions of SD rats on either low- or high-salt diets. They detected both renin enzymatic activity and angiotensinogen expression in these regions, showing that NaCl deprivation increased renin enzymatic activity in the olfactory bulb and anterior pituitary. The authors also controlled for residual plasma contamination by both perfusing rats and by evaluating renin enzymatic activity in brain regions of nephrectomized rats, experiments that collectively demonstrated the presence of *de novo* renin enzymatic activity in the CNS. These results corroborate previous findings from our group showing that renin and prorenin are expressed in the cortex, hypothalamus and brainstem of mice, and are elevated in the two latter brain regions of DOCA-salt hypertensive mice [24]. Because renin/sProrenin levels in the CNS are relatively low compared with those in the kidney, it has been a challenge to study its function. Notably, unlikely the case in the kidneys, it has never been shown that renin is stored in a type of granule cell in the brain, whereas it is predicted that sProrenin is secreted and acts on the PRR to exert its function [6, 73]. As very elegantly discussed in a recent review by Nakagawa *et al.* [73], the brain is composed of many neural circuitries, different cells and neuron types, and serves a large range of functions. Because RAS components may exhibit distinct distributions and expression in these brain regions, the cellular, neuroanatomical, and molecular specificity in the brain must be taken into consideration in making such determinations. Thus, techniques that are much more specific and sensitive than were used in these previous studies may be required to accurately assess renin/prorenin levels and function in the brain [73].

## 4 AN OVERVIEW OF THE PRR OUTSIDE THE CNS

The PRR, one of the more recently discovered members of the RAS, is a 350-amino-acid single-transmembrane protein composed of a large extracellular domain (ECD; ~310 amino acids), a single transmembrane domain (TMD; ~20 amino acids), and a short intracellular domain (ICD; ~19 amino acids) [6, 92]. The PRR forms homodimers through interactions of both the ECD and ICD [93-95] and is ubiquitously expressed, mainly in the plasma membrane but also in intracellular compartments like the perinuclear space [79, 92, 96, 97]. It is encoded by the *ATP6AP2* gene, localized on the X chromosome. The PRR exhibits a high degree of homology between species at the nucleotide sequence level (95%) and amino-acid sequence level (80%) [98]. PRR orthologs are present in both vertebrates and invertebrates. The ECD amino-acid sequence is similar among vertebrates, while the sequence of the ICD is highly conserved in vertebrates and invertebrates [99, 100]. The PRR has multiple functions, including activation of the tissue RAS [24, 70, 71, 75, 92, 101, 102] and activation of tyrosine phosphorylation-dependent signaling pathways [70, 75, 92, 102]. The PRR is also a component of the vacuolar H^+^-ATPase, acting as an adaptor protein between the H^+^-ATPase and the Wnt receptor complex, and plays an important role in lysosomal acidification and autophagy, among other functions [79]. In addition, an exonic splice enhancer mutation in the *ATP6AP2* gene leads to X-linked mental retardation and epilepsy, suggesting a novel role for the PRR in cognitive function and brain development [103]. Our focus here is on two PRR functions—mediating Ang II formation as a key RAS component, and promoting tyrosine phosphorylation-dependent signaling pathways—in BP regulation. For more information on these other PRR functions, see a recent review by Ichihara and Yatabe [79].

The PRR plays an important role in the activation of the tissue RAS. PRR binding to prorenin, previously considered to be an inactive renin precursor, leads to structural conformational changes in prorenin that lead to its activation without cleavage of the prosegment, suggesting that prorenin is important in the formation of Ang II in tissues where the PRR is expressed [6]. Binding either renin or prorenin to the PRR increases the catalytic efficiency of angiotensinogen cleavage to Ang I [92, 98]. Notably, when bound to the PRR, the catalytic efficiency of prorenin in converting angiotensinogen to Ang I is as high as that of renin [92]. Renin and prorenin binding to the PRR also induces intracellular signaling independent of the formation of Ang II, including activation of mitogen-activated protein kinases (MAPKs) such as p38 and c-Jun N-terminal kinase (JNK) [106], extracellular signal-regulated kinases (ERK)-1 and -2 (ERK2) [92, 104], as well as their downstream targets, heat shock protein 27 (HSP27) [105], transforming growth factor-β (TGFβ) and NADPH oxidase (NOX) [75, 106], enhancing the production of proinflammatory cytokines [107, 108] and expression of Wnt-mediated promyelocytic zinc finger (PLZF) protein [109].

Three different forms of the PRR have been described to date: the full length protein containing both N- and C- termini [92], a truncated form called the M8-9 fragment containing the C-terminal region [110], and a truncated form, also called soluble PRR (sPRR), containing the N-terminal region [111]. The M8-9 fragment and full-length PRR are associated with V-ATPase, which is essential for cell survival [110, 112]. Cousin *et al.* (2009) [111] showed that sPRR is cleaved by furin from the full-length PRR and is present in the circulation. They also showed that sPRR can bind both renin and prorenin. Several groups have investigated the physiological role of sPRR and its contribution to essential hypertension [113], obesity-induced hypertension [114, 115], pre-eclampsia [116], chronic heart failure [117, 118], and other health conditions. A recent study showed that circulating sPRR stimulates the RAS, increasing plasma renin concentration and angiotensinogen expression in the kidney and liver [114]. More recently, however, Wang and colleagues [119] showed that intravenous administration of histidine-tagged recombinant sPRR improves diet-induced obesity and metabolic syndrome. Despite several studies indicating that sPRR levels are increased in different pathophysiological conditions [120], as also discussed by Cousin *et al*. (2009) [111], it is yet not known whether sPRR can activate prorenin or if it competes with membrane full-length PRR, acting as a natural antagonist. Moreover, to our knowledge, no study to date has investigated whether sPRR plays a role in BP regulation through a central mechanism, a question that warrants further investigation.

As previously mentioned, the PRR is expressed in different organs and tissues, including the kidneys [92, 121], heart [92], brain [92], vascular smooth muscle cells [122], adipose tissue [123], placenta [92], liver [92] and pancreas [124], among others [125]. Specifically, several studies have investigated mechanisms related to the regulation of PRR expression in the kidney under different pathophysiological conditions. PRR expression in the kidney is upregulated by 1) glucose levels, through PKC-Raf-1-ERK1/2-NF-κB, PKC-JNK-NF-κB, and PKC-JNK-AP-1 (c-Jun) signaling pathways [126]; 2) sodium concentration, through xanthine oxidase activity in proximal tubules and mineralocorticoid receptor activation in distal tubules [127, 128]; 3) Ang II, through COX-2- [129] and AT_1_R-NADPH-dependent [130, 131] mechanisms; and 4) a high-fructose diet, through uric acid [132]. Nevertheless, our understanding of the mechanisms that regulate PRR expression in other tissues outside of the kidney is still very limited.

### 4.1 PRR expression in the CNS

PRR mRNA and protein are highly expressed in most regions of the mouse brain, including key regions involved in BP and body fluid regulation, such as the SFO, SON, RVLM, PVN and NTS [70, 96, 133-136], as also summarized in Table **1**. Based on the morphology of cells expressing PRR mRNA in the above-mentioned brain regions, Contrepas *et al.* [133], using *in situ* hybridization on paraffin-embedded mouse brain sections, reported in 2009 that the PRR is primarily expressed in neurons and cells of the choroid plexus. A year later, Shan and colleagues confirmed and extended these previous results in rats [134]. Using quantitative real-time PCR, they showed that the PRR is expressed in the brain regions indicated above, as well as the MnPO, OVLT and central amygdala (CA), and that it is expressed in neurons (NeuN^+^), but not astrocytes (GFAP^+^), in the SON. They also compared PRR mRNA expression in these brain regions between WKY rats and SHRs and reported that PRR expression is higher in the SON, CA, and NTS of SHR rats [134]. In 2012 and 2015, we and others demonstrated using immunofluorescence that the PRR is expressed in neurons of the mouse SFO, PVN, arcuate nucleus (Arc), NTS, RVLM, cortex and nucleus raphe pallidus [6, 70, 71, 74, 96], and that its expression level is higher in the SFO and PVN of Ang II-dependent [96] and DOCA-salt [137] hypertensive mice compared with normotensive mice. In the PVN, we found that the PRR is mostly expressed in neurons, but also in a few microglia cells [70]. Recently, Hu *et al*. (2020) [138] showed that both prorenin and PRR expression are increased in the RVLM in stress-induced hypertension. Takahashi *et al.* reported that, in humans, PRR mRNA is highly expressed in the CNS, including the hypothalamus and pituitary, and showed by immunohistochemistry that the PRR is expressed in the PVN and SON, where it is co-localized with arginine-vasopressin (AVP) and oxytocin in neurons in these nuclei [135]. Later, our group showed that the PRR is expressed in most neurons of the human SFO, with minor expression in microglia but no expression in astrocytes. Interestingly, PRR immunoreactivity is higher in this region in hypertensive humans [136], suggesting potential clinical significance. *In vitro*, two studies using cell culture techniques demonstrated the expression of the PRR in astrocytes and microglia from rodents. Specifically, Shan *et al.* reported that PRR mRNA is expressed in cultured astroglia from hypothalamic and brainstem regions of WKY rats [97]. In another study, Shi *et al.* (2014) [108] showed by immunolabeling that PRR protein is expressed in both cultured primary microglia from SD rats and in cultures of immortalized microglia (N9 cells) [108]. Overall, the PRR is mostly expressed in neurons *in vivo*, with minor expression in microglia, but not in astrocytes, although there is limited *in vitro* support for PRR expression in this latter cell type.

Our understanding of the mechanisms responsible for the regulation of PRR expression in the brain in both physiological and pathophysiological conditions remains very limited. Our group previously showed that PRR mRNA and protein levels are increased in the PVN and hypothalamus of DOCA-salt hypertensive mice and that treatment with losartan or captopril prevents the increase in PRR mRNA expression observed in these mice, indicating that Ang II/AT_1_R signaling regulates PRR expression [137]. Furthermore, this regulation of PRR expression is mediated by transcription factors downstream of the AT_1_R, namely cAMP response element-binding protein (CREB) and activator protein-1 (AP-1). Other factors and mechanisms that regulate the PRR in the CNS in various physiological and pathophysiological conditions, such as high salt or high-fat diet (HFD), remain unexplored.

In summary, these studies show that PRR mRNA and protein are mostly expressed in neurons of key brain cardiovascular regulatory regions in mice, rats, and humans. Some evidence supports the expression of the PRR in microglia, while no studies have shown PRR protein expression in astrocytes *in vivo*. Future studies using cell-specific reporter strains should provide additional insight into PRR expression patterns under physiological conditions and in the context of cardiovascular diseases, such as hypertension, heart failure, obesity, and diabetes. Furthermore, current knowledge regarding which neuronal types express the PRR is still limited for most brain regions. Advances in Cre-loxP technology, which uses the DNA recombinase, Cre, to either inactivate or induce the expression of a gene by genetic excision or inversion/translocation of DNA sequences between two loxP sites [139], should also facilitate investigation of PRR expression and function in different neuron types. In addition, future studies are warranted to understand how PRR expression in the CNS is regulated. This information will provide valuable insight into the pathogenesis of cardiovascular and metabolic diseases.

### 4.2 The PRR in CNS Regulation of BP: Gain-of-function Approaches

Only a few studies have investigated the role of the brain PRR in the regulation of BP using gain-of-function strategies, as summarized in Table **2**. In 2010, Shan *et al.* [134] induced human PRR overexpression in the SON of SD rats by microinjection of adeno-associated (AAV) virus encoding the human PRR (AAV-hPRR). They observed reductions in water intake and urine volume and osmolality, as well as increased levels of AVP in the urine and plasma, demonstrating the importance of the PRR in the SON in regulating fluid intake and AVP secretion. A few years later, a transgenic mouse expressing the hPRR specifically in neurons was developed and characterized [75]. These mice, in which hPRR is expressed under the control of the neuron-specific rat synapsin 1 promoter (Syn-hPRR mice), exhibit increased PRR expression in the SFO, PVN, NTS, AP, RVLM and other regions throughout the brain. Under baseline conditions, there is no difference in BP, HR, or locomotor activity between transgenic and wild-type (WT) mice; however, the pressor response to ICV infusion of human prorenin is greater in Syn-hPRR mice. Notably, the pressor response to prorenin infusion was not changed by co-infusion of the AT_1_R antagonist losartan or ACE inhibitor captopril, suggesting that the pressor response to prorenin is not caused by increased Ang II/AT_1_R signaling. In fact, Ang II levels are not increased in the hypothalamus of these mice. Interestingly, PRO20, a PRR antagonist, exerted a dose-dependent attenuation of the pressor response produced by the ICV infusion of prorenin. In this study, this pressor response was found to be mediated by ERK phosphorylation and NADPH oxidase 4 (NOX4) activation in the CNS. These data highlight the importance of Ang II-independent PRR signaling in the regulation of BP.

Several studies have also used gain-of-function approaches in *in vitro* cell culture systems to investigate the PRR and its mechanisms. Neuro-2A (N2A) cells overexpressing the human PRR show an increase in reactive oxygen species (ROS) production following incubation with mouse prorenin [102]. Interestingly, oxidative stress was attenuated by treating these cells with the AT_1_R antagonist losartan, suggesting that this effect, at least in part, is Ang II-dependent. In fact, incubation of these cells with prorenin resulted in both increased intracellular Ang II (whole-cell lysates) and secreted Ang II (in cell culture medium). There were also increases in the expression of NOX2 and NOX4 mRNAs in PRR-overexpressing N2A cells, suggesting a mechanism involving ROS production.

Results obtained by inducing hPRR overexpression in the SFO of mice by ICV injection of AAV2-hPRR virus provide additional support for this mechanism [102]. Specifically, PRR overexpression induced activation of PI3K and MAPK, resulting in NOX2 and NOX4 upregulation and increased ROS production. Notably, ROS production in PRR-overexpressing N2A cells was decreased by siRNA-mediated knockdown of NOX2 and NOX4. Taken together, these findings suggest that the binding of prorenin to the PRR induces oxidative stress in N2A cells, an effect that appears to be mediated by both Ang II-dependent and Ang II-independent pathways.

### 4.3 The PRR in CNS Regulation of BP: Genetic Loss-of- Function Approaches

Different approaches have been used to decrease PRR expression in studies investigating the role of PRR in the neural regulation of BP (Table **3**) [96, 107, 134]. Shan *et al.* (2010) [134] showed that PRR knockdown in the SON of SHRs induced by microinjection of AAV virus encoding shRNA against the PRR (AAV-PRR-shRNA) attenuated age-dependent increases in BP and decreased HR. These effects were associated with decreased levels of plasma AVP, revealing a role for the SON PRR in regulating BP and AVP secretion. In another study, Li *et al.* (2012) [96] knocked down the PRR in the SFO of hypertensive renin-angiotensinogen (RA) double-transgenic mice and found that PRR knockdown in the SFO resulted in decreased BP, improved spontaneous baroreflex sensitivity, and attenuated cardiac and vasomotor sympathetic tone. These effects were associated with a decrease in AT_1a_R mRNA and protein expression and AVP mRNA expression in the PVN as well as a decrease in plasma AVP levels, demonstrating the importance of the PRR in regulating autonomic function and AVP synthesis and secretion. Using a similar approach, Zubcevic *et al.* (2013) [107] knocked down the PRR in the NTS of 8-week-old SHRs and observed an increase in mean arterial pressure (MAP) and a shift in the cardiac baroreflex curve to higher MAP values, with no change in the maximum gain. These effects were associated with decreased mRNA expression of NF-κB (nuclear factor-κB), interleukin (IL)-6, tumor necrosis factor (TNF)-α, and C-C motif ligand 5 (Ccl5) in the NTS. They further investigated the cardiovascular effects of renin infusion in the NTS and showed that it produces a dose-dependent reduction in MAP and HR in SHRs, but not in WKY rats. Moreover, this effect was not changed by co-administration of losartan but was reversed by co-infusion of HRP, a PRR antagonist. Taken together, these data indicate that PRR activation has an excitatory effect on NTS neurons independent of Ang II.

The Cre-loxP system allows researchers to investigate the role of the PRR in specific cell types or specific brain regions in BP regulation. Li *et al.* (2014) [24] induced PRR knockdown specifically in neurons by breeding PRR-floxed mice with mice expressing Cre under the control of a pan neuronal promoter, the neuron filament heavy chain promoter (Nefh-Cre). In this study, PRR knockdown in neurons did not change baseline BP, HR, locomotor activity, or body weight. These mice exhibited expected pressor responses to the ICV infusion of carbachol (acetylcholine receptor agonist), Ang II, and mouse renin; interestingly, however, the pressor response to ICV-administered prorenin was reduced. These data indicate the importance of the PRR in mediating Ang II formation through activation of prorenin. Furthermore, in DOCA-salt hypertensive mice, PRR knockdown in neurons prevents the increased formation of Ang II and attenuates hypertension development and cardiac and vasomotor sympathetic tone, and improves cardiac parasympathetic tone. These results reveal the importance of the neuronal PRR in the formation of Ang II and the regulation of autonomic activity during hypertension.

In another study, neuronal PRR knockdown decreased saline and total fluid intake, sodium preference, urine volume and sodium excretion in response to DOCA, suggesting that the neuronal PRR has a regulatory role in sodium appetite [140]. More recently, it was shown that PRR knockdown in PVN neurons by microinjecting PRR-loxP mice with Cre-expressing AAV virus (AAV2-Cre) attenuated the development of DOCA-salt hypertension and improved autonomic function [70]. These effects were associated with 1) reduced phosphorylation of ERK1/2, a marker of neuronal activation, in PVN and RVLM neurons; 2) decreased AT_1a_R mRNA expression in the PVN; and 3) attenuated AT_1a_R-dependent Ca^2+^ activity in the PVN, providing a mechanistic basis for the role of the PRR in angiotensinergic signaling during DOCA-salt-induced hypertension. In a separate study, Pitra *et al.* (2019) [76] showed that PRR mRNA expression is also increased in the SON of DOCA-salt hypertensive rats. Using an electrophysiological approach, they further showed that the response to the application of prorenin was exacerbated in SON magnocellular neurons of hypertensive rats. Moreover, knockdown of the PRR selectively in neurons attenuated the elevation in plasma AVP levels observed in DOCA-salt hypertensive mice, again demonstrating a role for the PRR in AVP secretion. Finally, only one study to date has investigated the role of the PRR in the CNS in the pathogenesis of obesity-induced hypertension and type II diabetes [71]. In this recently published study, Worker *et al*. (2020) [71] showed that neuronal PRR deletion mediated by a pan-neuronal promoter (*Nefh*) attenuated the development of hypertension and type II diabetes induced by a 60% fat diet for 16 weeks. This dietary regimen was associated with marked increases in prorenin levels in the plasma and hypothalamus, as well as elevation of Ang II in the hypothalamus. Neuronal deletion of the PRR did not alter plasma Ang II or sProrenin levels in the plasma and hypothalamus, but did significantly reduce hypothalamic Ang II, supporting a critical role for the neuronal PRR in endogenous brain Ang II formation and metabolic regulation.

**Table 3:** Loss-of-function (LOF) approaches investigating the role of the brain PRR in the regulation of BP.

**Biological System**	**Cellular/Neuroanatomical** **Location**	**LOF Strategy**	**Main Results**	**Ang II-Dependent or Independent** **effects**	**Refs.**
Rat	SON	Microinjection of AAV-PRR-shRNA	• Decreases MAP, HR, and plasma AVP levels.	No	[134]
Mouse	SFO	ICV injection of AAV-PRR-shRNA	• Decreases MAP, vasomotor and cardiac sympathetic tone, and increases baroreflex sensitivity, and AVP level.	Reduction in AT_1_R	[96]
Rat	NTS	Microinjection of AAV-PRR-shRNA	• Increases MAP, impairs baroreflex sensitivity, and attenuates inflammation in SHR rats. • Renin microinjection decreases MAP and HR in SHR but not WKY rats. • Prorenin treatment in primary neurons induces NF-κB activation and increases mRNA expression of proinflammatory cytokines.	Ang II-independent cytokine signaling	[107]
Mouse	Neurons	Cre-loxp system: Deletion of PRR by Cre recombinase driven by pan neuronal neurofilament heavy chain promoter (Nefh-PRR KO)	• ICV infusion of mouse prorenin induces an elevation of BP that is blocked by neuronal PRR deletion. • PRR deletion decreases Ang II levels in the cortex, hypothalamus, and brainstem of DOCA-salt mice. • PRR knockdown attenuates the development of DOCA-salt hypertension and improves autonomic function.	Ang II formation in the CNS	[24]
Mouse	Neurons		• PRR deletion decreases salt appetite, fluid intake, urine volume, and sodium levels induced by DOCA.	No	[140]
Mouse	Neurons		• Prorenin acts *via* the PRR to increase the excitability of magnocellular neurons in the SON. • Prorenin-PRR signaling contributes to increased AVP levels in DOCA-salt mice.	No	[76]
Mouse	Neurons		• HFD increases prorenin levels in the plasma and hypothalamus. • HFD increases Ang II level in the hypothalamus; PRR deletion decreases Ang II levels in the hypothalamus. • PRR deletion attenuates obesity-induced hypertension and diabetes. • PRR deletion attenuates HFD-induced astrogliosis, and astrocytic and neuronal NF-κB p65 activation in the Arc.	Ang II formation in the CNS	[71]
Mouse	PVN neuron	Bilateral microinjection of AAV2-Cre virus into the PVN of PRR-loxP mice.	• PRR knockdown in PVN neurons attenuates the development of DOCA-salt hypertension. • PRR knockdown reduces ERK1/2 activation in PVN and RVLM neurons. • PRR knockdown reduces AT_1_ expression (mRNA) and attenuates AT_1_-dependent Ca^2+^ activity in the PVN.	Ang II-dependent and -independent ERK1/2 activation and Ca^2+^ activity	[70]

**Abbreviations:** AAV-PRR-shRNA, adeno-associated virus encoding shRNA against the PRR; AAV-Cre, adeno-associated virus encoding Cre recombinase; Ang II, angiotensin II; AT_1_, angiotensin II type 1 receptor; AVP, arginine vasopressin; DOCA, deoxycorticosterone; ERK1/2, extracellular signal-regulated kinase 1/2; HFD, high-fat diet; HR, heart rate; HTN, hypertensive; ICV, intracerebroventricular; MAP, mean arterial pressure; mRenin, mouse renin; NF-κB, nuclear factor-κB; NTS, nucleus tractus solitarius; PVN, paraventricular nucleus; RA, renin angiotensinogen mice; RVLM, rostral ventrolateral medulla; SFO, subfornical organ; SHR, spontaneously hypertensive rat; SON, supraoptic nucleus; WKY, Wistar Kyoto rat; WT, wild type.

### 4.4 PRR in the CNS Regulation of BP: Pharmacological Approaches

One of the strategies that has advanced our understanding of the role the CNS PRR plays in BP regulation is the central administration of exogenous prorenin. ICV infusion of prorenin increases BP; this effect is attenuated by PRR deletion [24] in neurons and exacerbated by neuronal PRR overexpression [75]. Furthermore, PVN microinjection of prorenin increases sympathetic splanchnic nerve activity [74]. Collectively, these finds indicate that prorenin acts through the PRR to mediate its effects. Only a few studies have used PRR antagonists to investigate the role of the brain PRR in the regulation of BP. To date, two PRR antagonists, HRP [141] and PRO20 [25], have been developed and used in these studies. HRP is a decoy peptide containing the handle region of the prorenin prosegment and inhibits the conformational change and non-proteolytic activation of prorenin induced by its binding to the PRR [141, 142]. Treatment with HRP attenuates the development of diabetic nephropathy [141, 143], decreases glomerulosclerosis in aged rats [106], diminishes cardiac hypertrophy in hypertensive rats [144], and improves fat distribution in obese mice [145]. However, some studies have suggested that HRP does not inhibit prorenin binding to the PRR [146] and might instead have a partial agonistic effect on the PRR, which may be part of the reason for the conflicting results obtained using this decoy peptide [6, 146-149]. On the other hand, PRO20, composed of the first 20 amino acids of the prorenin prosegment, is a competitive antagonist of prorenin binding to the PRR [6, 25]. It has been shown that treatment with PRO20 decreases BP and attenuates kidney injury in hypertensive mice [150], decreases BP in a model of high fructose-induced salt sensitivity [132], reduces high-salt-induced apoptosis in inner medullary collecting duct cells [151], and mitigates protein overload-induced renal injury [152]. In addition, PRO20 has been used as a tool to better understand the role of the PRR in renal physiology [153-157], including the role of the collecting duct PRR in regulating aquaporin 2 expression [155] and epithelial sodium channel (ENaC) activity [154], and the role of the renal PRR in potassium homeostasis [156].

Focusing on the CNS, Zubcevic *et al.* (2013) showed that infusion of renin into the NTS of SHR rats decreased MAP and improved the baroreflex sensitivity of these rats, effects that were reversed by co-administration of HRP [107]. Another group has also used HRP to investigate the effects of prorenin on microglia cell cultures [108]. These researchers showed that prorenin has proinflammatory effects in these cells and that these effects are attenuated by incubation with HRP. On the other hand, PRO20 blocks the binding of prorenin to both mouse and human PRRs in the brain and prevents prorenin-induced Ca^2+^ influx in human neuronal cells [25]. Moreover, ICV infusion of PRO20 attenuates the pressor effects of ICV-infused prorenin in normotensive mice and lowers BP in mice with either Ang II-dependent hypertension or DOCA-salt hypertension. ICV infusion of PRO20 also decreases cardiac and vasomotor sympathetic tone and increases cardiac parasympathetic tone in DOCA-salt mice. Consistent with these findings, ICV infusion of PRO20 reduces the formation of Ang II in the cortex, hypothalamus, and brainstem of DOCA-salt mice [25]. Recently, Hu *et al*. (2020) [138] reported that rats with stress-induced hypertension exhibit higher BP and renal sympathetic nerve activity (RSNA) as well as increased expression of prorenin and the PRR in the RVLM. Chronic intracisternal infusion of PRO20 into these rats decreased BP and RSNA. Mechanistically, these researchers found that a proinflammatory state developed in the RVLM of SIH rats, with increased microglia proliferation and activation, NLRP3 expression in microglia, ROS production, and levels of IL-1β and TNF-α. Interestingly, treatment with PRO20 attenuated these changes. Taken together, these studies show that PRR antagonism is a new therapeutic approach that could be used to study physiological and pathophysiological roles of the PRR and as a new pharmacological approach for the treatment of cardio-renal and metabolic diseases.

## CONCLUSION AND PERSPECTIVE

While our understanding of the role of the brain PRR in the regulation of BP has advanced considerably, the importance of the brain PRR in other cardiovascular and metabolic diseases remains undefined. As summarized in Fig. (**1**), the PRR exerts an excitatory role in key cardiovascular regulatory regions of the brain and is involved in the regulation of inflammation, neuronal activity, autonomic function, sodium appetite, and AVP synthesis and secretion. These effects are mediated through both Ang II-dependent and -independent signaling pathways, as well as activation of oxidative stress, PI3K, MAPK, and NF-κB signaling pathways. Although the PRR is expressed mainly in neurons in the CNS, the mechanisms regulating the neuronal PRR and neural circuitries of the PRR in specific neuron types remain largely undefined. With advances in the Cre-loxP system, future development of a PRR-Cre mouse strain and cell-specific reporter strains can be expected, providing important tools to better understand the role and mechanisms regulating PRR expression under physiological conditions and in the pathogenesis of different cardiovascular diseases, including, but not limited to, hypertension, heart failure, and diabetes. The precise cellular localization and functional importance of CNS sProrenin, the endogenous ligand for the brain PRR, is another critical knowledge gap that warrants further investigation.

The clinical significance of the PRR is beginning to emerge through accumulating basic and translational research. Traditional RAS blockers such as angiotensin receptor blockers and ACE inhibitors induce secretion of reactive renin, increasing its levels and enzymatic activity in plasma [158-164]. Furthermore, plasma renin levels are elevated by treatment with aliskiren [165], a renin inhibitor used for the treatment of hypertension. However, aliskiren does not inhibit the binding of renin or prorenin to the PRR or activation of direct PRR signaling pathways [166]. Therefore, the indirect actions of these drugs might result in clinical consequences owing to activation of Ang II-independent PRR signaling, a possibility that needs further evaluation. Future studies investigating Ang II-dependent and/or -independent brain PRR signaling pathways in various cardiovascular and metabolic diseases would provide critical evidence supporting the clinical relevance of the PRR. In the end, by inhibiting both tissue/local RAS overactivation and Ang II-independent direct PRR signal pathways, PRR antagonism might present a better strategy for controlling BP and end-organ damage. Additional PRR antagonists would thus be valuable tools for advancing our understanding of the role of the PRR in a wide range of cardiovascular, metabolic and aging-related diseases, and could ultimately constitute a new category of drugs for treating these diseases.

Although the role of sPRR in BP regulation has been investigated in several peripheral tissues, its potential role in neural autonomic regulation remains unexplored and needs further investigation. Among other functions, the PRR is a component of the vacuolar H^+^-ATPase, acting as an adaptor protein between the H^+^-ATPase and the Wnt receptor complex to play an important role in lysosomal acidification and autophagy. However, no studies have yet investigated whether these PRR functions play a role in the neural regulation of BP. Although additional discoveries await, the takeaway point from the literature is that the PRR is a key component of the brain RAS that mediates endogenous Ang II formation and Ang II -independent signaling pathways.

## Figures and Tables

**Fig. (1):**
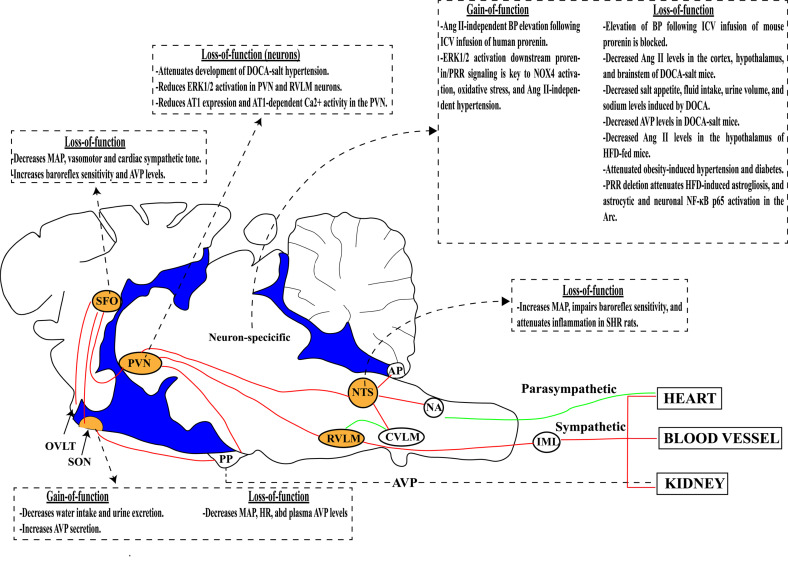
**Schematic depiction of PRR action in brain regions that regulate BP**. In the CNS, the PRR exerts an excitatory function in the subfornical organ (SFO), supraoptic nucleus (SON), paraventricular nucleus (PVN) and nucleus tractus solitarius (NTS; orange) and is involved in the regulation of autonomic function and arginine vasopressin (AVP) synthesis and secretion. Dashed arrows and boxes summarize the main findings by PRR gain- and/or loss-of-function approaches in specific brain regions or in CNS neurons. AP, area postrema; AT_1_, angiotensin II type I receptor; BP, blood pressure; CVLM, caudal ventrolateral medulla; DOCA, deoxycorticosterone; HFD, high-fat diet; IML, intermediolateral column; MAP, mean arterial pressure; NA, nucleus ambiguus; PP, posterior pituitary; RVLM, rostral ventrolateral medulla.

**Table 1:** Studies investigating PRR expression in the CNS.

**Biological System**	**Cellular/Neuroanatomical Location**	**Main Methods**	**PRR mRNA and Protein Expression**	**Refs.**
Cell culture	Neurons and astrocytes from the hypothalamus and brainstem of WKY rats	RT-qPCR, IF	mRNA in astrocytes; mRNA and protein in neurons from hypothalamic and brainstem regions	[97]
Mouse	SFO, PVN, SON, NTS, and RVLM	ISH	mRNA in the SFO, PVN, SON, NTS, and RVLM	[133]
Rat	SON, CA, and NTS	Brain micropunch, RT-qPCR, IF	mRNA levels increased in the SON, CA, and NTS of SHRs; Protein mainly detected in neurons of the SON	[134]
Human postmortem brain	PVN and SON	RT-qPCR, IHC	mRNA in the PVN and SON; Protein in vasopressin-positive and oxytocin-positive neurons	[135]
Mouse	SFO, PVN, NTS, RVLM, and NPR	IF	Protein in neurons of the SFO, PVN, NTS, RVLM, and NPR; Protein level elevated in the SFO and PVN of hypertensive RA mice	[96]
Cell culture	Immortalized cell line (N-9 mouse microglia cells), primary microglia from rats	IF	Protein in microglia	[108]
Mouse	PVN/hypothalamus	RT-qPCR, WB	mRNA level increased in the PVN of DOCA-salt mice; Protein level increased in the hypothalamus of DOCA-salt mice	[137]
Rat	PVN	IF	Protein in neurons but not astrocytes	[74]
Human postmortem brain	SFO	IF	Protein in neurons and microglia, but not astrocytes; Protein level elevated in hypertensive humans, and positively correlated with BP	[136]
Mouse	PVN	IF	Protein mainly in neurons and a few microglia, but not in astrocytes	[70]
Mouse	Arc	IF	Protein is expressed in neurons but not astrocytes or microglia	[71]
Rat	RVLM	RT-qPCR, WB	PRR and prorenin mRNA and protein increased in the RVLM of SIH rats	[138]

**Abbreviations:** Arc, arcuate nucleus; CA, central amygdala; IF, immunofluorescence; IHC, immunohistochemistry; ISH, *in situ* hybridization; PVN, paraventricular nucleus; NPR, nucleus of raphe pallidus; NTS, nucleus tractus solitarius; RA, renin angiotensinogen mice; RT-qPCR, quantitative reverse transcription PCR; RVLM, rostral ventrolateral medulla; SFO, subfornical organ; SHR, spontaneously hypertensive rats; SIH, stress-induced hypertension; SON, supraoptic nucleus; WB, Western blotting; WKY, Wistar Kyoto rats.

**Table 2:** Gain-of-function (GOF) approaches investigating the role of the brain PRR in the regulation of BP.

**Biological System**	**Cellular/Neuroanatomical** **Location**	**GOF Strategy**	**Main Results**	**Ang II-Dependent or Independent** **Effects Investigated?**	**Refs.**
Rat	SON WKY brain neurons	Microinjection of AAV-hPRR.	• hPRR overexpression decreases water intake and urine excretion. • hPRR overexpression increases urine osmolality, plasma and urine AVP levels.	Ang II formation in primary culture WKY brain neurons	[134]
Cell culture	Neuro-2A cells	Infected N2A cells with AAV-hPRR	• hPRR overexpression increases Ang II levels, ERK1/2 and Akt phosphorylation, and ROS. • PI3K and MAPK inhibition attenuate oxidative stress caused by hPRR overexpression.	Ang II generation and Ang II-independent MAPK signal pathways	[102]
Mouse	All neurons	Transgenic overexpression of hPRR driven by rat synapsin1 promoter	• ICV infusion of human prorenin induces Ang II-independent BP elevation. • ERK1/2 activation downstream of prorenin/PRR signaling is key to NOX4 activation, oxidative stress and ANG II-independent hypertension.	Ang II-independent ERK1/2, NOX4, and oxidative stress activation	[75]

**Abbreviations:** AAV-hPRR, adeno-associated virus encoding human PRR; Akt, protein kinase B; Ang II, angiotensin II; AVP, arginine vasopressin; ERK1/2, signal-regulated kinase 1/2; ICV, intracerebroventricular; MAPK, mitogen-activated protein kinase; N2A, Neuro-2A cells; NOX, NADPH oxidase; NF-κB, nuclear factor-κB; PI3K, phosphoinositide 3-kinase; PVN, paraventricular nucleus; RA, renin angiotensinogen mice; ROS, reactive oxygen species; SD, Sprague-Dawley; SHR, spontaneously hypertensive rats; SON, supraoptic nucleus.

## References

[1] Paul M., Poyan Mehr A., Kreutz R. (2006). Physiology of local renin-angiotensin systems.. Physiol. Rev..

[2] Ibrahim M.M. (2006). RAS inhibition in hypertension.. J. Hum. Hypertens..

[3] Mann S.J. (2003). Neurogenic essential hypertension revisited: The case for increased clinical and research attention.. Am. J. Hypertens..

[4] Hall J.E. (1986). Control of sodium excretion by angiotensin II: Intrarenal mechanisms and blood pressure regulation.. Am. J. Physiol..

[5] Crowley S.D., Zhang J., Herrera M., Griffiths R., Ruiz P., Coffman T.M. (2011). Role of AT receptor-mediated salt retention in angiotensin II-dependent hypertension.. Am. J. Physiol. Renal Physiol..

[6] Zhou Y., Chen Y., Dirksen W.P., Morris M., Periasamy M. (2003). AT1b receptor predominantly mediates contractions in major mouse blood vessels.. Circ. Res..

[7] Mulrow P.J., Ganong W.F. (1961). Stimulation of aldosterone secretion by angiotensisn. II. A preliminary report.. Yale J. Biol. Med..

[8] Tanabe A., Naruse M., Arai K., Naruse K., Yoshimoto T., Seki T., Imaki T., Kobayashi M., Miyazaki H., Demura H. (1998). Angiotensin II stimulates both aldosterone secretion and DNA synthesis* via *type 1 but not type 2 receptors in bovine adrenocortical cells.. J. Endocrinol. Invest..

[9] Naruse M, Tanabe A, Sato A (2002). Aldosterone breakthrough during angiotensin II receptor antagonist therapy in stroke-prone spontaneously hypertensive rats.. Hypertension (Dallas, Tex : 1979).

[10] Tsuda K. (2012). Renin-Angiotensin system and sympathetic neurotransmitter release in the central nervous system of hypertension.. Int. J. Hypertens..

[11] Fisher J.P., Paton J.F. (2012). The sympathetic nervous system and blood pressure in humans: Implications for hypertension.. J. Hum. Hypertens..

[12] Ferrario CM (1983). Neurogenic actions of angiotensin II.. Hypertension (Dallas, Tex : 1979).

[13] Zimmerman C.A., Lin Y.C., Leib D.E., Guo L., Huey E.L., Daly G.E., Chen Y., Knight Z.A. (2016). Thirst neurons anticipate the homeostatic consequences of eating and drinking.. Nature.

[14] Bader M. (2010). Tissue renin-angiotensin-aldosterone systems: Targets for pharmacological therapy.. Annu. Rev. Pharmacol. Toxicol..

[15] Santos R.A.S., Sampaio W.O., Alzamora A.C., Motta-Santos D., Alenina N., Bader M., Campagnole-Santos M.J. (2018). The ACE2/angiotensin-(1-7)/mas axis of the renin-angiotensin system: focus on angiotensin-(1-7).. Physiol. Rev..

[16] Xu Q., Jensen D.D., Peng H., Feng Y. (2016). The critical role of the central nervous system (pro)renin receptor in regulating systemic blood pressure.. Pharmacol. Ther..

[17] Miyata S. (2015). New aspects in fenestrated capillary and tissue dynamics in the sensory circumventricular organs of adult brains.. Front. Neurosci..

[18] Hendel M.D., Collister J.P. (2005). Contribution of the subfornical organ to angiotensin II-induced hypertension.. Am. J. Physiol. Heart Circ. Physiol..

[19] Biancardi V.C., Stern J.E. (2016). Compromised blood-brain barrier permeability: Novel mechanism by which circulating angiotensin II signals to sympathoexcitatory centres during hypertension.. J. Physiol..

[20] Ganten D., Minnich J.L., Granger P., Hayduk K., Brecht H.M., Barbeau A., Boucher R., Genest J. (1971). Angiotensin-forming enzyme in brain tissue.. Science.

[21] Schelling P., Hutchinson J.S., Ganten U., Sponer G., Ganten D. (1976). Impermeability of the blood-cerebrospinal fluid barrier for angiotensin II in rats.. Clin. Sci. Mol. Med. Suppl..

[22] Nakagawa P, Sigmund CD (2017). How is the brain renin-angiotensin system regulated?. Hypertension.

[23] Karamyan V.T., Speth R.C. (2007). Enzymatic pathways of the brain renin-angiotensin system: Unsolved problems and continuing challenges.. Regul. Pept..

[24] Li W., Peng H., Mehaffey E.P., Kimball C.D., Grobe J.L., van Gool J.M., Sullivan M.N., Earley S., Danser A.H., Ichihara A., Feng Y. (2014). Neuron-specific (pro)renin receptor knockout prevents the development of salt-sensitive hypertension.. Hypertension.

[25] Li W., Sullivan M.N., Zhang S., Worker C.J., Xiong Z., Speth R.C., Feng Y. (2015). Intracerebroventricular infusion of the (Pro)renin receptor antagonist PRO20 attenuates deoxycorticosterone acetate-salt-induced hypertension.. Hypertension.

[26] Pires P.W., Dams C.M., Matin N., Dorrance A.M. (2013). The effects of hypertension on the cerebral circulation.. Am. J. Physiol. Heart Circ. Physiol..

[27] Biancardi V.C., Son S.J., Ahmadi S., Filosa J.A., Stern J.E. (2014). Circulating angiotensin II gains access to the hypothalamus and brain stem during hypertension* via *breakdown of the blood-brain barrier.. Hypertension.

[28] Dzau V.J., Ingelfinger J., Pratt R.E., Ellison K.E. (1986). Identification of renin and angiotensinogen messenger RNA sequences in mouse and rat brains.. Hypertension.

[29] Lavoie JL, Cassell MD, Gross KW, Sigmund CD (2004). Adjacent expression of renin and angiotensinogen in the rostral ventrolateral medulla using a dual-reporter transgenic model.. Hypertension.

[30] Stornetta R.L., Hawelu-Johnson C.L., Guyenet P.G., Lynch K.R. (1988). Astrocytes synthesize angiotensinogen in brain.. Science.

[31] Thomas W.G., Sernia C. (1988). Immunocytochemical localization of angiotensinogen in the rat brain.. Neuroscience.

[32] Ohkubo H., Nakayama K., Tanaka T., Nakanishi S. (1986). Tissue distribution of rat angiotensinogen mRNA and structural analysis of its heterogeneity.. J. Biol. Chem..

[33] Hirose S., Naruse M., Ohtsuki K., Inagami T. (1981). Totally inactive renin zymogen and different forms of active renin in hog brain tissues.. J. Biol. Chem..

[34] Chai S.Y., Mendelsohn F.A., Paxinos G. (1987). Angiotensin converting enzyme in rat brain visualized by quantitative *in vitro* autoradiography.. Neuroscience.

[35] Rogerson F.M., Schlawe I., Paxinos G., Chai S.Y., McKinley M.J., Mendelsohn F.A. (1995). Localization of angiotensin converting enzyme by *in vitro* autoradiography in the rabbit brain.. J. Chem. Neuroanat..

[36] Strittmatter S.M., Lo M.M., Javitch J.A., Snyder S.H. (1984). Autoradiographic visualization of angiotensin-converting enzyme in rat brain with [3H]captopril: Localization to a striatonigral pathway.. Proc. Natl. Acad. Sci. USA.

[37] Song K., Allen A.M., Paxinos G., Mendelsohn F.A. (1992). Mapping of angiotensin II receptor subtype heterogeneity in rat brain.. J. Comp. Neurol..

[38] Tsutsumi K., Saavedra J.M. (1991). Quantitative autoradiography reveals different angiotensin II receptor subtypes in selected rat brain nuclei.. J. Neurochem..

[39] Tsutsumi K., Saavedra J.M. (1991). Characterization and development of angiotensin II receptor subtypes (AT1 and AT2) in rat brain.. Am. J. Physiol..

[40] Johren O., Inagami T., Saavedra J.M. (1995). AT1A, AT1B, and AT2 angiotensin II receptor subtype gene expression in rat brain.. Neuroreport.

[41] Zhuo J., Moeller I., Jenkins T., Chai S.Y., Allen A.M., Ohishi M., Mendelsohn F.A. (1998). Mapping tissue angiotensin-converting enzyme and angiotensin AT1, AT2 and AT4 receptors.. J. Hypertens..

[42] Allen A.M., Zhuo J., Mendelsohn F.A. (2000). Localization and function of angiotensin AT1 receptors.. Am. J. Hypertens..

[43] de Kloet A.D., Wang L., Pitra S., Hiller H., Smith J.A., Tan Y., Nguyen D., Cahill K.M., Sumners C., Stern J.E., Krause E.G. (2017). A unique “Angiotensin-Sensitive” neuronal population coordinates neuroendocrine, cardiovascular, and behavioral responses to stress.. J. Neurosci..

[44] de Kloet A.D., Wang L., Ludin J.A., Smith J.A., Pioquinto D.J., Hiller H., Steckelings U.M., Scheuer D.A., Sumners C., Krause E.G. (2016). Reporter mouse strain provides a novel look at angiotensin type-2 receptor distribution in the central nervous system.. Brain Struct. Funct..

[45] Lenkei Z., Palkovits M., Corvol P., Llorens-Cortes C. (1996). Distribution of angiotensin II type-2 receptor (AT2) mRNA expression in the adult rat brain.. J. Comp. Neurol..

[46] Bunnemann B., Iwai N., Metzger R., Fuxe K., Inagami T., Ganten D. (1992). The distribution of angiotensin II AT1 receptor subtype mRNA in the rat brain.. Neurosci. Lett..

[47] Sumners C, Alleyne A, Rodríguez V (2019). Brain angiotensin type-1 and type-2 receptors: Cellular locations under normal and hypertensive conditions.. Hypertens Res.

[48] Xia H., Lazartigues E. (2008). Angiotensin-converting enzyme 2 in the brain: Properties and future directions.. J. Neurochem..

[49] Donoghue M., Hsieh F., Baronas E., Godbout K., Gosselin M., Stagliano N., Donovan M., Woolf B., Robison K., Jeyaseelan R., Breitbart R.E., Acton S. (2000). A novel angiotensin-converting enzyme-related carboxypeptidase (ACE2) converts angiotensin I to angiotensin 1-9.. Circ. Res..

[50] Feng Y., Yue X., Xia H., Bindom S.M., Hickman P.J., Filipeanu C.M., Wu G., Lazartigues E. (2008). Angiotensin-converting enzyme 2 overexpression in the subfornical organ prevents the angiotensin II-mediated pressor and drinking responses and is associated with angiotensin II type 1 receptor downregulation.. Circ. Res..

[51] Feng Y., Xia H., Cai Y., Halabi C.M., Becker L.K., Santos R.A., Speth R.C., Sigmund C.D., Lazartigues E. (2010). Brain-selective overexpression of human Angiotensin-converting enzyme type 2 attenuates neurogenic hypertension.. Circ. Res..

[52] Becker LK, Etelvino GM, Walther T, Santos RA, Campagnole-Santos MJ (2007). Immunofluorescence localization of the receptor Mas in cardiovascular-related areas of the rat brain.. Am J Physiol Heart Circ Physiol..

[53] Santos R.A.S., Simoes e Silva A.C., Maric C., Silva D.M., Machado R.P., de Buhr I., Heringer-Walther S., Pinheiro S.V., Lopes M.T., Bader M., Mendes E.P., Lemos V.S., Campagnole-Santos M.J., Schultheiss H.P., Speth R., Walther T. (2003). Angiotensin-(1-7) is an endogenous ligand for the G protein-coupled receptor Mas.. Proc. Natl. Acad. Sci. USA.

[54] Santos R.A. (2014). Angiotensin-(1-7).. Hypertension.

[55] Rabelo LA, Alenina N, Bader M (2011). ACE2-angiotensin-(1-7)-Mas axis and oxidative stress in cardiovascular disease.. Hypertension Res.

[56] Samani N.J., Swales J.D., Brammar W.J. (1988). Expression of the renin gene in extra-renal tissues of the rat.. Biochem. J..

[57] Iwai N., Inagami T. (1992). Quantitative analysis of renin gene expression in extrarenal tissues by polymerase chain reaction method.. J. Hypertens..

[58] Lavoie J.L., Cassell M.D., Gross K.W., Sigmund C.D. (2004). Localization of renin expressing cells in the brain, by use of a REN-eGFP transgenic model.. Physiol. Genomics.

[59] Allen A.M., O’Callaghan E.L., Hazelwood L., Germain S., Castrop H., Schnermann J., Bassi J.K. (2008). Distribution of cells expressing human renin-promoter activity in the brain of a transgenic mouse.. Brain Res..

[60] Jones C.A., Hurley M.I., Black T.A., Kane C.M., Pan L., Pruitt S.C., Gross K.W. (2000). Expression of a renin/GFP transgene in mouse embryonic, extra-embryonic, and adult tissues.. Physiol. Genomics.

[61] Grobe J.L., Xu D., Sigmund C.D. (2008). An intracellular renin-angiotensin system in neurons: Fact, hypothesis, or fantasy.. Physiology.

[62] Sinn P.L., Sigmund C.D. (2000). Identification of three human renin mRNA isoforms from alternative tissue-specific transcriptional initiation.. Physiol. Genomics.

[63] Lee-Kirsch M.A., Gaudet F., Cardoso M.C., Lindpaintner K. (1999). Distinct renin isoforms generated by tissue-specific transcription initiation and alternative splicing.. Circ. Res..

[64] Jackson L., Eldahshan W., Fagan S.C., Ergul A. (2018). Within the brain: The renin angiotensin system.. Int. J. Mol. Sci..

[65] Lenz T., Sealey J.E., Maack T., James G.D., Heinrikson R.L., Marion D., Laragh J.H. (1991). Half-life, hemodynamic, renal, and hormonal effects of prorenin in cynomolgus monkeys.. Am. J. Physiol..

[66] Lavoie J.L., Liu X., Bianco R.A., Beltz T.G., Johnson A.K., Sigmund C.D. (2006). Evidence supporting a functional role for intracellular renin in the brain.. Hypertension.

[67] Shinohara K., Liu X., Morgan D.A., Davis D.R., Sequeira-Lopez M.L., Cassell M.D., Grobe J.L., Rahmouni K., Sigmund C.D. (2016). Selective deletion of the brain-specific isoform of renin causes neurogenic hypertension.. Hypertension.

[68] Xu D., Borges G.R., Davis D.R., Agassandian K., Sequeira Lopez M.L., Gomez R.A., Cassell M.D., Grobe J.L., Sigmund C.D. (2011). Neuron- or glial-specific ablation of secreted renin does not affect renal renin, baseline arterial pressure, or metabolism.. Physiol. Genomics.

[69] Shinohara K., Nakagawa P., Gomez J., Morgan D.A., Littlejohn N.K., Folchert M.D., Weidemann B.J., Liu X., Walsh S.A., Ponto L.L., Rahmouni K., Grobe J.L., Sigmund C.D. (2017). Selective deletion of renin-b in the brain alters drinking and metabolism.. Hypertension.

[70] Souza L.A.C., Worker C.J., Li W., Trebak F., Watkins T., Gayban A.J.B., Yamasaki E., Cooper S.G., Drumm B.T., Feng Y. (2019). (Pro)renin receptor knockdown in the paraventricular nucleus of the hypothalamus attenuates hypertension development and AT_1_ receptor-mediated calcium events.. Am. J. Physiol. Heart Circ. Physiol..

[71] Worker C.J., Li W., Feng C.Y., Souza L.A.C., Gayban A.J.B., Cooper S.G., Afrin S., Romanick S., Ferguson B.S., Feng Earley Y. (2020). The neuronal (pro)renin receptor and astrocyte inflammation in the central regulation of blood pressure and blood glucose in mice fed a high-fat diet.. Am. J. Physiol. Endocrinol. Metab..

[72] Xu J., Sriramula S., Xia H., Moreno-Walton L., Culicchia F., Domenig O., Poglitsch M., Lazartigues E. (2017). Clinical relevance and role of neuronal at_1_ receptors in adam17-mediated ace2 shedding in neurogenic hypertension.. Circ. Res..

[73] Nakagawa P., Gomez J., Grobe J.L., Sigmund C.D. (2020). The renin-angiotensin system in the central nervous system and its role in blood pressure regulation.. Curr. Hypertens. Rep..

[74] Huber M.J., Basu R., Cecchettini C., Cuadra A.E., Chen Q.H., Shan Z. (2015). Activation of the (pro)renin receptor in the paraventricular nucleus increases sympathetic outflow in anesthetized rats.. Am. J. Physiol. Heart Circ. Physiol..

[75] Peng H., Jensen D.D., Li W., Sullivan M.N., Buller S.A., Worker C.J., Cooper S.G., Zheng S., Earley S., Sigmund C.D., Feng Y. (2018). Overexpression of the neuronal human (pro)renin receptor mediates angiotensin II-independent blood pressure regulation in the central nervous system.. Am. J. Physiol. Heart Circ. Physiol..

[76] Pitra S., Worker C.J., Feng Y., Stern J.E. (2019). Exacerbated effects of prorenin on hypothalamic magnocellular neuronal activity and vasopressin plasma levels during salt-sensitive hypertension.. Am. J. Physiol. Heart Circ. Physiol..

[77] Grobe J.L., Rahmouni K., Liu X., Sigmund C.D. (2013). Metabolic rate regulation by the renin-angiotensin system: Brain *vs.* body.. Pflugers Arch..

[78] Genain C.P., Van Loon G.R., Kotchen T.A. (1985). Distribution of renin activity and angiotensinogen in rat brain. Effects of dietary sodium chloride intake on brain renin.. J. Clin. Invest..

[79] Ichihara A., Yatabe M.S. (2019). The (pro)renin receptor in health and disease.. Nat. Rev. Nephrol..

[80] van Thiel BS, Góes Martini A, Te Riet L (2017). Brain renin-angiotensin system: does it exist?. Hypertension (Dallas, Tex: 1979).

[81] Batenburg W.W., Lu X., Leijten F., Maschke U., Müller D.N., Danser A.H. (2011). Renin- and prorenin-induced effects in rat vascular smooth muscle cells overexpressing the human (pro)renin receptor: Does (pro)renin-(pro)renin receptor interaction actually occur?. Hypertension.

[82] Sevá Pessôa B., van der Lubbe N., Verdonk K., Roks A.J., Hoorn E.J., Danser A.H. (2013). Key developments in renin-angiotensin-aldosterone system inhibition.. Nat. Rev. Nephrol..

[83] Danser A.H.J. (2015). The role of the (Pro)renin receptor in hypertensive disease.. Am. J. Hypertens..

[84] Sigmund C.D., Diz D.I., Chappell M.C. (2017). No brain renin-angiotensin system.. Hypertension.

[85] Hirose S., Yokosawa H., Inagami T. (1978). Immunochemical identification of renin in rat brain and distinction from acid proteases.. Nature.

[86] Hermann K., Raizada M.K., Sumners C., Phillips M.I. (1987). Presence of renin in primary neuronal and glial cells from rat brain.. Brain Res..

[87] Sakai K., Agassandian K., Morimoto S., Sinnayah P., Cassell M.D., Davisson R.L., Sigmund C.D. (2007). Local production of angiotensin II in the subfornical organ causes elevated drinking.. J. Clin. Invest..

[88] Schinke M., Baltatu O., Böhm M., Peters J., Rascher W., Bricca G., Lippoldt A., Ganten D., Bader M. (1999). Blood pressure reduction and diabetes insipidus in transgenic rats deficient in brain angiotensinogen.. Proc. Natl. Acad. Sci. USA.

[89] Baltatu O., Silva J.A., Ganten D., Bader M. (2000). The brain renin-angiotensin system modulates angiotensin II-induced hypertension and cardiac hypertrophy.. Hypertension.

[90] Itaya Y., Suzuki H., Matsukawa S., Kondo K., Saruta T. (1986). Central renin-angiotensin system and the pathogenesis of DOCA-salt hypertension in rats.. Am. J. Physiol..

[91] Park C.G., Leenen F.H. (2001). Effects of centrally administered losartan on deoxycorticosterone-salt hypertension rats.. J. Korean Med. Sci..

[92] Nguyen G., Delarue F., Burcklé C., Bouzhir L., Giller T., Sraer J.D. (2002). Pivotal role of the renin/prorenin receptor in angiotensin II production and cellular responses to renin.. J. Clin. Invest..

[93] Zhang Y., Gao X., Michael Garavito R. (2011). Structural analysis of the intracellular domain of (pro)renin receptor fused to maltose-binding protein.. Biochem. Biophys. Res. Commun..

[94] Suzuki-Nakagawa C., Nishimura M., Tsukamoto T., Aoyama S., Ebihara A., Suzuki F., Nakagawa T. (2014). Participation of the extracellular domain in (pro)renin receptor dimerization.. Biochem. Biophys. Res. Commun..

[95] Schefe J.H., Menk M., Reinemund J., Effertz K., Hobbs R.M., Pandolfi P.P., Ruiz P., Unger T., Funke-Kaiser H. (2006). A novel signal transduction cascade involving direct physical interaction of the renin/prorenin receptor with the transcription factor promyelocytic zinc finger protein.. Circ. Res..

[96] Li W., Peng H., Cao T., Sato R., McDaniels S.J., Kobori H., Navar L.G., Feng Y. (2012). Brain-targeted (pro)renin receptor knockdown attenuates angiotensin II-dependent hypertension.. Hypertension.

[97] Shan Z., Cuadra A.E., Sumners C., Raizada M.K. (2008). Characterization of a functional (pro)renin receptor in rat brain neurons.. Exp. Physiol..

[98] Nguyen G., Muller D.N. (2010). The biology of the (pro)renin receptor.. J. Am. Soc. Nephrol..

[99] Burcklé C., Bader M. (2006). Prorenin and its ancient receptor.. Hypertension.

[100] Yang T. (2017). Unraveling the Physiology of (Pro)Renin Receptor in the Distal Nephron.. Hypertension.

[101] Nurun N.A., Uddin N.M., Nakagawa T., Iwata H., Ichihara A., Inagami T., Suzuki F. (2007). Role of “handle” region of prorenin prosegment in the non-proteolytic activation of prorenin by binding to membrane anchored (pro)renin receptor.. Front. Biosci..

[102] Peng H., Li W., Seth D.M., Nair A.R., Francis J., Feng Y. (2013). (Pro)renin receptor mediates both angiotensin II-dependent and -independent oxidative stress in neuronal cells.. PLoS One.

[103] Ramser J., Abidi F.E., Burckle C.A., Lenski C., Toriello H., Wen G., Lubs H.A., Engert S., Stevenson R.E., Meindl A., Schwartz C.E., Nguyen G. (2005). A unique exonic splice enhancer mutation in a family with X-linked mental retardation and epilepsy points to a novel role of the renin receptor.. Hum. Mol. Genet..

[104] Feng Y. (2015). ANG II-independent prorenin/(pro)renin receptor signaling pathways in the central nervous system.. Am. J. Physiol. Heart Circ. Physiol..

[105] Saris J.J., ’t Hoen P.A., Garrelds I.M., Dekkers D.H., den Dunnen J.T., Lamers J.M., Jan Danser A.H. (2006). Prorenin induces intracellular signaling in cardiomyocytes independently of angiotensin II.. Hypertension.

[106] Kaneshiro Y., Ichihara A., Sakoda M., Takemitsu T., Nabi A.H., Uddin M.N., Nakagawa T., Nishiyama A., Suzuki F., Inagami T., Itoh H. (2007). Slowly progressive, angiotensin II-independent glomerulosclerosis in human (pro)renin receptor-transgenic rats.. J. Am. Soc. Nephrol..

[107] Zubcevic J., Jun J.Y., Lamont G., Murça T.M., Shi P., Yuan W., Lin F., Carvajal J.M., Li Q., Sumners C., Raizada M.K., Shan Z. (2013). Nucleus of the solitary tract (pro)renin receptor-mediated antihypertensive effect involves nuclear factor-κB-cytokine signaling in the spontaneously hypertensive rat.. Hypertension.

[108] Shi P., Grobe J.L., Desland F.A., Zhou G., Shen X.Z., Shan Z., Liu M., Raizada M.K., Sumners C. (2014). Direct pro-inflammatory effects of prorenin on microglia.. PLoS One.

[109] Cruciat C.M., Ohkawara B., Acebron S.P., Karaulanov E., Reinhard C., Ingelfinger D., Boutros M., Niehrs C. (2010). Requirement of prorenin receptor and vacuolar H+-ATPase-mediated acidification for Wnt signaling.. Science.

[110] Ludwig J., Kerscher S., Brandt U., Pfeiffer K., Getlawi F., Apps D.K., Schägger H. (1998). Identification and characterization of a novel 9.2-kDa membrane sector-associated protein of vacuolar proton-ATPase from chromaffin granules.. J. Biol. Chem..

[111] Cousin C., Bracquart D., Contrepas A., Corvol P., Muller L., Nguyen G. (2009). Soluble form of the (pro)renin receptor generated by intracellular cleavage by furin is secreted in plasma.. Hypertension.

[112] Kinouchi K., Ichihara A., Sano M., Sun-Wada G.H., Wada Y., Kurauchi-Mito A., Bokuda K., Narita T., Oshima Y., Sakoda M., Tamai Y., Sato H., Fukuda K., Itoh H. (2010). The (pro)renin receptor/ATP6AP2 is essential for vacuolar H+-ATPase assembly in murine cardiomyocytes.. Circ. Res..

[113] Morimoto S, Ando T, Niiyama M (2014). Serum soluble (pro)renin receptor levels in patients with essential hypertension.. Hypertension Res.

[114] Gatineau E., Cohn D.M., Poglitsch M., Loria A.S., Gong M., Yiannikouris F. (2019). Losartan prevents the elevation of blood pressure in adipose-PRR deficient female mice while elevated circulating sPRR activates the renin-angiotensin system.. Am. J. Physiol. Heart Circ. Physiol..

[115] Gatineau E., Gong M.C., Yiannikouris F. (2019). Soluble prorenin receptor increases blood pressure in high fat-fed male mice.. Hypertension.

[116] Mikami Y., Takai Y., Narita T., Era S., Ono Y., Saitoh M., Baba K., Matsuoka K., Seki H. (2017). Associations between the levels of soluble (pro)renin receptor in maternal and umbilical cord blood and hypertensive disorder of pregnancy.. Placenta.

[117] Obradovic D., Loncar G., Radenovic S., Tahirovic E., Heidecke H., Schulze-Forster K., Muller D., Busjahn A., Buttner P., Veskovic J., Zdravkovic M., Li H., Li S., Savkovic V., Pieske B., Dungen H.D., Dechend R. (2020). Soluble (pro)renin receptor in elderly chronic heart failure patients.. Front. Biosci..

[118] Gong L., Zhang S., Li L., Gao X., Wang D., Wu D., Wang K., Liu Y. (2019). Elevated plasma soluble (pro)renin receptor levels are associated with left ventricular remodeling and renal function in chronic heart failure patients with reduced ejection fraction.. Peptides.

[119] Wang F., Luo R., Zou C.J., Xie S., Peng K., Zhao L., Yang K.T., Xu C., Yang T. (2020). Soluble (pro)renin receptor treats metabolic syndrome in mice with diet-induced obesity* via *interaction with PPARγ.. JCI Insight.

[120] Zhu Q., Yang T. (2018). Enzymatic sources and physio-pathological functions of soluble (pro)renin receptor.. Curr. Opin. Nephrol. Hypertens..

[121] Riediger F., Quack I., Qadri F., Hartleben B., Park J.K., Potthoff S.A., Sohn D., Sihn G., Rousselle A., Fokuhl V., Maschke U., Purfürst B., Schneider W., Rump L.C., Luft F.C., Dechend R., Bader M., Huber T.B., Nguyen G., Muller D.N. (2011). Prorenin receptor is essential for podocyte autophagy and survival.. J. Am. Soc. Nephrol..

[122] Kurauchi-Mito A, Ichihara A, Bokuda K (2014). Significant roles of the (pro)renin receptor in integrity of vascular smooth muscle cells.. Hypertension research : Official journal of the Japanese Society of Hypertension.

[123] Achard V., Boullu-Ciocca S., Desbriere R., Nguyen G., Grino M. (2007). Renin receptor expression in human adipose tissue.. Am. J. Physiol. Regul. Integr. Comp. Physiol..

[124] Dai F.F., Bhattacharjee A., Liu Y., Batchuluun B., Zhang M., Wang X.S., Huang X., Luu L., Zhu D., Gaisano H., Wheeler M.B. (2015). A novel GLP1 receptor interacting protein ATP6ap2 regulates insulin secretion in pancreatic beta cells.. J. Biol. Chem..

[125] Ramkumar N., Kohan D.E. (2019). The (pro)renin receptor: An emerging player in hypertension and metabolic syndrome.. Kidney Int..

[126] Huang J., Siragy H.M. (2010). Regulation of (pro)renin receptor expression by glucose-induced mitogen-activated protein kinase, nuclear factor-kappaB, and activator protein-1 signaling pathways.. Endocrinology.

[127] Rong R., Ito O., Mori N., Muroya Y., Tamura Y., Mori T., Ito S., Takahashi K., Totsune K., Kohzuki M. (2015). Expression of (pro)renin receptor and its upregulation by high salt intake in the rat nephron.. Peptides.

[128] Yamakoshi S., Ito O., Rong R., Ohsaki Y., Nakamura T., Hirose T., Takahashi K., Mori T., Totsune K., Kohzuki M. (2020). High salt intake-increased (pro)renin receptor expression is exaggerated in the kidney of dahl salt-sensitive rats.. Hypertension.

[129] Wang F., Lu X., Peng K., Zhou L., Li C., Wang W., Yu X., Kohan D.E., Zhu S.F., Yang T. (2014). COX-2 mediates angiotensin II-induced (pro)renin receptor expression in the rat renal medulla.. Am. J. Physiol. Renal Physiol..

[130] Siragy H.M., Huang J. (2008). Renal (pro)renin receptor upregulation in diabetic rats through enhanced angiotensin AT1 receptor and NADPH oxidase activity.. Exp. Physiol..

[131] Gonzalez A.A., Womack J.P., Liu L., Seth D.M., Prieto M.C. (2014). Angiotensin II increases the expression of (pro)renin receptor during low-salt conditions.. Am. J. Med. Sci..

[132] Xu C, Lu A, Lu X (2017). Activation of renal (Pro)renin receptor contributes to high fructose-induced salt sensitivity.. Hypertension (Dallas, Tex : 1979).

[133] Contrepas A., Walker J., Koulakoff A., Franek K.J., Qadri F., Giaume C., Corvol P., Schwartz C.E., Nguyen G. (2009). A role of the (pro)renin receptor in neuronal cell differentiation.. Am. J. Physiol. Regul. Integr. Comp. Physiol..

[134] Shan Z., Shi P., Cuadra A.E., Dong Y., Lamont G.J., Li Q., Seth D.M., Navar L.G., Katovich M.J., Sumners C., Raizada M.K. (2010). Involvement of the brain (pro)renin receptor in cardiovascular homeostasis.. Circ. Res..

[135] Takahashi K., Hiraishi K., Hirose T., Kato I., Yamamoto H., Shoji I., Shibasaki A., Kaneko K., Satoh F., Totsune K. (2010). Expression of (pro)renin receptor in the human brain and pituitary, and co-localisation with arginine vasopressin and oxytocin in the hypothalamus.. J. Neuroendocrinol..

[136] Cooper S.G., Trivedi D.P., Yamamoto R., Worker C.J., Feng C.Y., Sorensen J.T., Yang W., Xiong Z., Feng Y. (2018). Increased (pro)renin receptor expression in the subfornical organ of hypertensive humans.. Am. J. Physiol. Heart Circ. Physiol..

[137] Li W., Liu J., Hammond S.L., Tjalkens R.B., Saifudeen Z., Feng Y. (2015). Angiotensin II regulates brain (pro)renin receptor expression through activation of cAMP response element-binding protein.. Am. J. Physiol. Regul. Integr. Comp. Physiol..

[138] Hu L., Zhang S., Ooi K., Wu X., Wu J., Cai J., Sun Y., Wang J., Zhu D., Chen F., Xia C. (2020). Microglia-derived NLRP3 activation mediates the pressor effect of prorenin in the rostral ventrolateral medulla of stress-induced hypertensive rats.. Neurosci. Bull..

[139] Kim H., Kim M., Im S.K., Fang S. (2018). Mouse Cre-LoxP system: General principles to determine tissue-specific roles of target genes.. Lab. Anim. Res..

[140] Trebak F., Li W., Feng Y. (2018). Neuronal (pro)renin receptor regulates deoxycorticosterone-induced sodium intake.. Physiol. Genomics.

[141] Ichihara A., Hayashi M., Kaneshiro Y., Suzuki F., Nakagawa T., Tada Y., Koura Y., Nishiyama A., Okada H., Uddin M.N., Nabi A.H., Ishida Y., Inagami T., Saruta T. (2004). Inhibition of diabetic nephropathy by a decoy peptide corresponding to the “handle” region for nonproteolytic activation of prorenin.. J. Clin. Invest..

[142] Suzuki F., Hayakawa M., Nakagawa T., Nasir U.M., Ebihara A., Iwasawa A., Ishida Y., Nakamura Y., Murakami K. (2003). Human prorenin has “gate and handle” regions for its non-proteolytic activation.. J. Biol. Chem..

[143] Ichihara A., Sakoda M., Kurauchi-Mito A., Kaneshiro Y., Itoh H. (2008). Involvement of (pro)renin receptor in the glomerular filtration barrier.. J. Mol. Med. (Berl.).

[144] Wu J., Zhang C., Liu C., Zhang A., Li A., Zhang J., Zhang Y. (2019). Aortic constriction induces hypertension and cardiac hypertrophy* via *(pro)renin receptor activation and the PLC-β3 signaling pathway.. Mol. Med. Rep..

[145] Tan P., Blais C., Nguyen T.M.D., Schiller P.W., Gutkowska J., Lavoie J.L. (2016). Prorenin/renin receptor blockade promotes a healthy fat distribution in obese mice.. Obesity (Silver Spring).

[146] Feldt S, Batenburg WW, Mazak I (2008). Prorenin and renin-induced extracellular signal-regulated kinase 1/2 activation in monocytes is not blocked by aliskiren or the handle-region peptide.. Hypertension.

[147] Batenburg W.W., van den Heuvel M., van Esch J.H.M., van Veghel R., Garrelds I.M., Leijten F., Danser A.H. (2013). The (pro)renin receptor blocker handle region peptide upregulates endothelium-derived contractile factors in aliskiren-treated diabetic transgenic (mREN2)27 rats.. J. Hypertens..

[148] van Esch JHM, van Veghel R, Garrelds IM (2011). Handle region peptide counteracts the beneficial effects of the Renin inhibitor aliskiren in spontaneously hypertensive rats.. Hypertension.

[149] Krebs C., Weber M., Steinmetz O., Meyer-Schwesinger C., Stahl R., Danser A.H., Garrelds I., van Goor H., Nguyen G., Müller D., Wenzel U. (2008). Effect of (pro)renin receptor inhibition by a decoy peptide on renal damage in the clipped kidney of Goldblatt rats.. Kidney Int..

[150] Wang F., Lu X., Liu M., Feng Y., Zhou S.F., Yang T. (2015). Renal medullary (pro)renin receptor contributes to angiotensin II-induced hypertension in rats* via *activation of the local renin-angiotensin system.. BMC Med..

[151] Su J., Liu X., Xu C., Lu X., Wang F., Fang H., Lu A., Qiu Q., Li C., Yang T. (2017). NF-κB-dependent upregulation of (pro)renin receptor mediates high-NaCl-induced apoptosis in mouse inner medullary collecting duct cells.. Am. J. Physiol. Cell Physiol..

[152] Fang H., Deng M., Zhang L., Lu A., Su J., Xu C., Zhou L., Wang L., Ou J.S., Wang W., Yang T. (2018). Role of (pro)renin receptor in albumin overload-induced nephropathy in rats.. Am. J. Physiol. Renal Physiol..

[153] Lu X., Wang F., Liu M., Yang K.T., Nau A., Kohan D.E., Reese V., Richardson R.S., Yang T. (2016). Activation of ENaC in collecting duct cells by prorenin and its receptor PRR: Involvement of Nox4-derived hydrogen peroxide.. Am. J. Physiol. Renal Physiol..

[154] Peng K., Lu X., Wang F., Nau A., Chen R., Zhou S.F., Yang T. (2017). Collecting duct (pro)renin receptor targets ENaC to mediate angiotensin II-induced hypertension.. Am. J. Physiol. Renal Physiol..

[155] Wang F., Lu X., Peng K., Fang H., Zhou L., Su J., Nau A., Yang K.T., Ichihara A., Lu A., Zhou S.F., Yang T. (2016). Antidiuretic action of collecting duct (pro)renin receptor downstream of vasopressin and pge2 receptor ep4.. J. Am. Soc. Nephrol..

[156] Xu C., Fang H., Zhou L., Lu A., Yang T. (2016). High potassium promotes mutual interaction between (pro)renin receptor and the local renin-angiotensin-aldosterone system in rat inner medullary collecting duct cells.. Am. J. Physiol. Cell Physiol..

[157] Xu C., Lu A., Wang H., Fang H., Zhou L., Sun P., Yang T. (2017). (Pro)Renin receptor regulates potassium homeostasis through a local mechanism.. Am. J. Physiol. Renal Physiol..

[158] Belz G.G., Butzer R., Kober S., Mutschler E. (2002). Pharmacodynamic studies on the angiotensin II type 1 antagonists irbesartan and candesartan based on angiotensin II dose response in humans.. J. Cardiovasc. Pharmacol..

[159] Jones M.R., Sealey J.E., Laragh J.H. (2007). Effects of angiotensin receptor blockers on ambulatory plasma Renin activity in healthy, normal subjects during unrestricted sodium intake.. Am. J. Hypertens..

[160] Maillard M.P., Würzner G., Nussberger J., Centeno C., Burnier M., Brunner H.R. (2002). Comparative angiotensin II receptor blockade in healthy volunteers: The importance of dosing.. Clin. Pharmacol. Ther..

[161] Azizi M., Ménard J., Bissery A., Guyenne T.T., Bura-Rivière A., Vaidyanathan S., Camisasca R.P. (2004). Pharmacologic demonstration of the synergistic effects of a combination of the renin inhibitor aliskiren and the AT1 receptor antagonist valsartan on the angiotensin II-renin feedback interruption.. J. Am. Soc. Nephrol..

[162] Juillerat L., Nussberger J., Ménard J., Mooser V., Christen Y., Waeber B., Graf P., Brunner H.R. (1990). Determinants of angiotensin II generation during converting enzyme inhibition.. Hypertension.

[163] Collier J.G., Jenkins J.S., Keddie J., Khan M.U., Robinson B.F. (1974). Effect of angiotensin-converting enzyme inhibitor on response of plasma renin activity and aldosterone to tilting in man.. Br. J. Clin. Pharmacol..

[164] Larochelle P., Gutkowska J., Schiffrin E., Kuchel O., Hamet P., Genest J. (1985). Effect of enalapril on renin, angiotensin converting enzyme activity, aldosterone and prostaglandins in patients with hypertension.. Clin. Invest. Med..

[165] Sealey J.E., Laragh J.H. (2007). Aliskiren, the first renin inhibitor for treating hypertension: Reactive renin secretion may limit its effectiveness.. Am. J. Hypertens..

[166] Schefe J.H., Neumann C., Goebel M., Danser J., Kirsch S., Gust R., Kintscher U., Unger T., Funke-Kaiser H. (2008). Prorenin engages the (pro)renin receptor like renin and both ligand activities are unopposed by aliskiren.. J. Hypertens..

